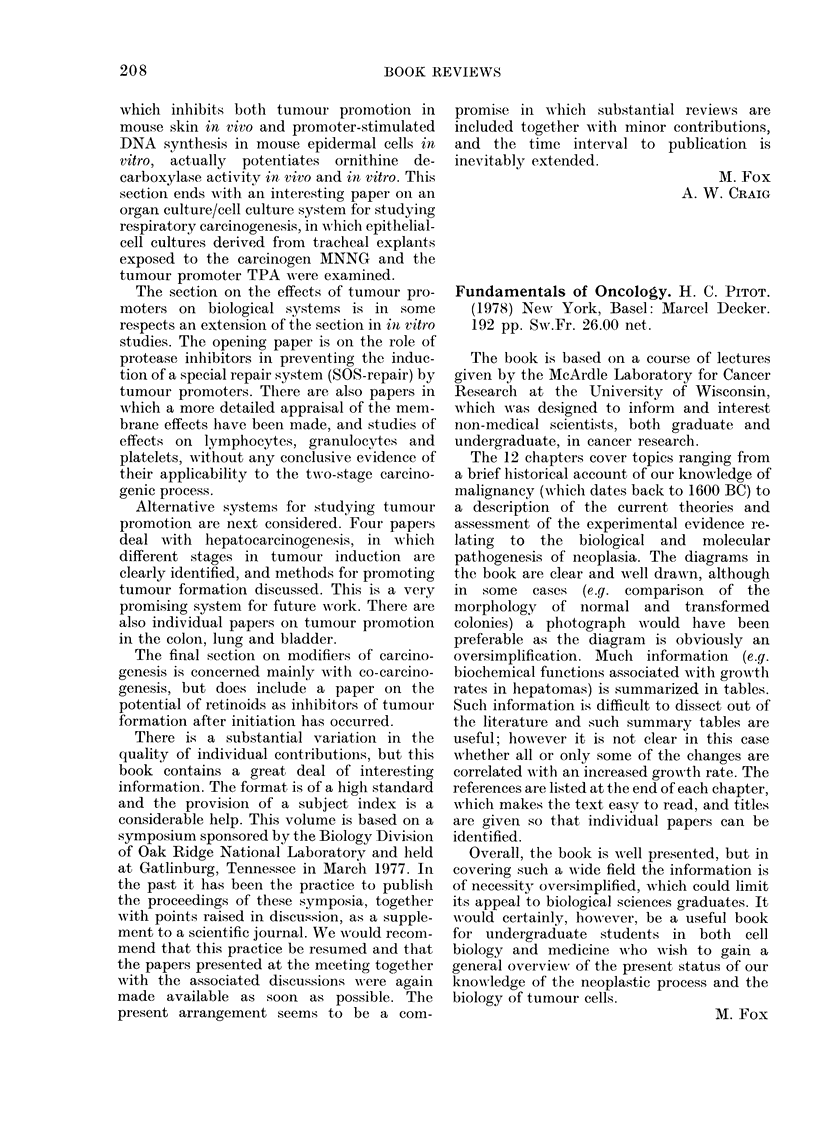# Fundamentals of Oncology

**Published:** 1979-02

**Authors:** M. Fox


**Fundamentals of Oncology.** H. C. Pitot. (1978) New York, Basel: Marcel Decker. 192 pp. Sw.Fr. 26.00 net.

The book is based on a course of lectures given by the McArdle Laboratory for Cancer Research at the University of Wisconsin, which was designed to inform and interest non-medical scientists, both graduate and undergraduate, in cancer research.

The 12 chapters cover topics ranging from a brief historical account of our knowledge of malignancy (which dates back to 1600 BC) to a description of the current theories and assessment of the experimental evidence relating to the biological and molecular pathogenesis of neoplasia. The diagrams in the book are clear and well drawn, although in some cases (*e.g.* comparison of the morphology of normal and transformed colonies) a photograph would have been preferable as the diagram is obviously an oversimplification. Much information (*e.g.* biochemical functions associated with growth rates in hepatomas) is summarized in tables. Such information is difficult to dissect out of the literature and such summary tables are useful; however it is not clear in this case whether all or only some of the changes are correlated with an increased growth rate. The references are listed at the end of each chapter, which makes the text easy to read, and titles are given so that individual papers can be identified.

Overall, the book is well presented, but in covering such a wide field the information is of necessity oversimplified, which could limit its appeal to biological sciences graduates. It would certainly, however, be a useful book for undergraduate students in both cell biology and medicine who wish to gain a general overview of the present status of our knowledge of the neoplastic process and the biology of tumour cells.

M. Fox